# Creating Ambassadors of Planet Earth: The Overview Effect in K12 Education

**DOI:** 10.3389/fpsyg.2020.540996

**Published:** 2020-10-07

**Authors:** H. Anna T. van Limpt - Broers, Marie Postma, Max M. Louwerse

**Affiliations:** Department of Cognitive Science and Artificial Intelligence, Tilburg University, Tilburg, Netherlands

**Keywords:** overview effect, virtual reality, education, immersion, awe

## Abstract

The overview effect is the commonly reported experience of astronauts viewing planet Earth from space and the subsequent reflection on and processing of this experience. The overview effect is associated with feelings of awe, self-transcendence, and a change of perspective and identity that manifest themselves in taking steps toward protecting the fragile ecosystem. In the current study, we investigated whether the overview effect can be obtained in school children when simulated using virtual reality (VR) and whether the effect has a positive impact on learning gains. Using questionnaires and attention data in an existing simulation environment used in the school system, we showed that the VR simulation elicits an overview effect experience. Moreover, the experience yields learning gains in the domain of astrophysics. These findings are in line with past evidence regarding the positive impact of awe on learning and can be used to support further investigations of the relation between the overview effect and behavioral changes, specifically for educational purposes.

## Introduction

Since the 50th anniversary of the landing on the Moon and the first photograph of planet Earth outside its atmosphere, a renewed interest has emerged in the intense experience reported by astronauts. This experience was dubbed as the overview effect (White, 2014), an overwhelming experience when viewing the Earth from space and the subsequent reflection on and processing of this experience (Nezami, 2017). White (2014) describes the overview effect as a cognitive shift in awareness caused by seeing Earth protected by a very-thin-looking ozone layer in the hostility of space. Interestingly, the overview effect is not experienced uniformly, with personality traits, such as a need for cognition and religiousness, playing a role (Gallagher et al., 2014). In general, though, the effect appears to be associated with feelings of *compassion* and *self-transcendence*, a change of *perspective* and *identity*, and *awe* (Yaden et al., 2016). Some astronauts consider it a spiritual experience leading to self-transcendence (Nelson-Coffey et al., 2019), a “temporary feeling of unity characterized by reduced self-salience and increased feelings of connection” (Yaden et al., 2016).

The overview effect can also give rise to an increased understanding of how life interconnects, a renewed motivation to protect the planet’s environment, and a feeling of kinship with people across the globe (Ihle et al., 2006) as well as a strong feeling of compassion toward others. Meanwhile, recognizing familiar locations on Earth from outside its orbit strengthens the feeling of personal connection (Stepanova et al., 2019b). Experiencing the overview effect leads to a lasting increase in the appreciation and the concern for planet Earth and a connection with humanity. This can support more pro-social attitudes such as a sense of international unity and altruistic behaviors (Yaden et al., 2016).

Since the anecdotal evidence in White (2014), the overview effect has been studied in the lab as well, with numerous studies demonstrating that it is a reproducible experience. The first and most important group of experimental participants consisted of astronauts. Among others, Ihle et al. (2006), Kanas (2020), and Nezami (2017) investigated the positive effects that spaceflight has on the well-being of people who had been in space, including aspects of the overview effect relating to the beauty and the fragility of Earth and the unity of mankind. Gallagher et al. (2014) analyzed astronaut journals and post-flight reports and compared the described experiences to the reports of participants of a virtual recreation of viewing Earth from space. The comparison revealed that the overview effect can be constructed, at least to some extent, also in simulated environments. When trying to recreate the overview effect in people on Earth, it is vital that they experience a feeling of awe, a key concept of the overview effect (Stepanova et al., 2019a).

While most accounts of the overview effect rely on self-reports, there is some evidence that measurements of brain activity may provide additional information. Arguably, the feelings associated with the overview effect can be compared to meditation in experienced meditators, i.e., clarity of thought and a change in perception of space and time. Meditation-like experiences can be measured not only through self-reports but also through the measurement of brain activity (Newberg et al., 2001; Berkovich-Ohana et al., 2013). Recently, it has been shown that meditation-like experiences can be simulated in virtual reality (VR), bringing participants in an immersive environment while recording neurophysiological responses (Tinga et al., 2019). Tinga et al. (2019) found brain imaging evidence for meditation in EEG theta to alpha ratios. Similarly, Gallagher et al. (2014) reported differences in theta and beta activity throughout the brain for those who experienced a simulated overview effect. In their experiment, they let the participants experience an environment of the International Space Station with portals opened to display simulations of Earth or deep space.

Brain activity is not the only objective measure that can be used to investigate the overview effect. Stepanova et al. (2019a) suggest that gaze data (even lower-resolution gaze data from a VR headset) could be used as possible indicators of the experience since an awed viewer is not able to tear their eyes away from the object(s) triggering the effect. As astronaut Scott Carpenter reports in White(2014, p. 29): “I found it difficult to tear my eyes away and go on to something else. Everything is so new and awe-inspiring that it is difficult to concentrate for very long on any one thing.” Next to that, smoothness of the gaze movement, as well as longer dwell time, can suggest a calm gaze pattern associated with the effect.

Some studies suggest that the overview effect is mediated by awe. Awe appears to be a prominent feature associated with the experience and an important feeling to achieve while recreating an overview effect-inducing experience in VR (Stepanova et al., 2019a). Awe can be defined as a feeling of being overwhelmed and impressed by greatness or vastness (Keltner and Haidt, 2003), for example, open and rugged scenes (Klatzky et al., 2017) that elicit the need for cognitive accommodation. Vastness includes impressive views as well as an understanding of complex theory (Chirico and Yaden, 2018). Seeing something from a very high vantage point thus coincides with an experience of vastness. White (2014) mentions on the first page of his book that “anyone who flies in an airplane and looks out the window has the opportunity to experience a mild version of (the Overview Effect).” This indicates its connection to awe. Other environments and occurrences that can elicit the same type of emotions are cathedrals (Keltner and Haidt, 2003), (videos of) natural panoramic views and scenes (Rudd et al., 2012; Van Cappellen and Saroglou, 2012; van Elk et al., 2016; Guan et al., 2019; McPhetres, 2019), childbirth (Van Cappellen and Saroglou, 2012; Shiota et al., 2017), natural disasters (Keltner and Haidt, 2003; Guan et al., 2019), and, of course, videos of Earth from space (Rudd et al., 2012; Nelson-Coffey et al., 2019). Similarly to the overview effect, awe-inducing events can result in a feeling of self-transcendence and transformation as well as spirituality (Van Cappellen and Saroglou, 2012; Chirico and Yaden, 2018). It can also make one feel small (van Elk et al., 2016), increase pro-social behavior, and support integration into social groups (Piff et al., 2015; Bai et al., 2017). Awe also plays an important role in learning. In particular, a high propensity for awe has been shown to support the adaptability of mental schemas (Shiota et al., 2007b) and thus the openness to learn from awe-inspiring experiences or “surprising discoveries” (Valdesolo et al., 2017; Gottlieb et al., 2018). Particularly, young children learn much more effectively when their belief about the world is disproved, resulting in a need to accommodate the experience (Stahl and Feigenson, 2017). Awe has also been linked to greater awareness of knowledge gaps. That is, awe has been shown to make gaps in one’s knowledge salient, which could be attributed to it being a positive emotion (McPhetres, 2019). These findings open up the question on whether the overview effect can be simulated for elementary school children, instilling the feeling of awe in them, and support learning. The first goal of the current study was to establish the relation between two types of cognitive states, compassion and awe, to the overview effect recreated in VR and to explore their impact on potential learning gains.

Whether or not strong feelings of awe will be achieved in immersive environments created by VR (Chirico et al., 2017) depends on the degree of immersion. A higher degree means a greater approximation of realism (Bangay and Preston, 1998). High immersion in VR has been reported to improve learning and memory; however, the importance of measuring presence when examining the effect of immersion on performance is critical (de Back et al., 2018). Presence describes the degree of felt realism and of “being there” (Witmer and Singer, 1998). Mikropoulos and Strouboulis (2004) reported effects of presence in 12-year-old children in an educational VR, with these effects having a positive effect on engagement and motivation. However, it should be noted that, although VR simulations have been reported to motivate and interest students and increase their performance in some studies (Alhalabi, 2016; de Back et al., 2018), other studies did not confirm these findings (Parong and Mayer, 2018). Since presence increases the feeling of actually being in a situation, such as viewing the Earth from space, we would expect presence to influence the overview effect (Stepanova et al., 2019b). Similar to presence, immersive tendencies refer to an individual’s likelihood to experience presence (Witmer and Singer, 1998). Therefore, the second goal of the current study was to test the impact of immersive tendencies and presence on the overview effect experience and on the achieved learning gains.

To address both goals, the overview effect experience was created in VR in collaboration with SpaceBuzz. SpaceBuzz is a non-profit organization, located in the Netherlands, that offers an innovative educational program consisting of a pre-flight and a post-flight training including a simulated rocket launch in VR to 10–12-year-old children. In the pre-flight program, children write an application letter to become an astronaut, put puzzles together using oven mitts as a proxy for space suit gloves, and hang upside down on a playground bar while eating their lunch to familiarize themselves with gravity. After the children pass the astronaut training, they are launched into virtual space inside of an actual rocket ship. Using 4D simulations, children are sent into orbit around planet Earth, guided on their trip by a virtual embodiment of the ESA astronaut André Kuipers. After returning from their VR trip to Earth, a post-flight program at school has children give press conferences to friends and family, just like real astronauts.

## Current Study

The aim of the current study was to investigate whether we would be able to recreate the overview effect in VR for children between 10 and 12 years and examine its impact on learning. Based on previous findings, we predicted the relation to be affected by compassion, awe, and presence.

### Method

#### Participants

In total, 233 children from eight classes in six schools in the Netherlands participated in the study. Of these 233 participants, 45 participants were removed from the analysis because of 40 questionnaires not being returned and five pre-tests being filled out at the wrong time (15% of the participants) (age *M* = 10.68, SD = 0.70; 87 boys, 93 girls, eight unreported).

### Procedure

Prior to the study, parents received a letter that explained the nature of the experiment. Of the children who were allowed to participate, children with medical issues or those being worried about being dizzy or nauseous participated in the VR simulation under the direct supervision of their teacher. The study was approved by the Ethical Review Board of Tilburg University (REDC #2019/04a).

In the six participating schools, teachers were informed about the educational program and the VR experience onsite. The pre-flight program consisted of either four or six lessons. All children received the same theoretical and creative classes about the universe, planets, and satellites, received identical questionnaires on paper, and filled them out individually.

For the VR flight program, the classes traveled to the SpaceBuzz rocketship at a location close to the schools. They experienced the simulation in groups of nine participants. SpaceBuzz attendants provided a brief explanation about how the Head Mounted Display had to be worn and asked the children to keep their hands on their knees. If the children placed their hands too far to the side, the attendant placed them back on their lap for safety. After completing the VR experience, the children filled out questionnaires at a separate table under the supervision of research assistants.

In the schools, teachers taught the six post-flight lessons about worlds without borders and science. Some remaining questionnaires were administered.

### Materials

#### Pre-flight

The pre-flight program included several questionnaires and learning activities. The first was a personality questionnaire, which was a writing exercise where children chose three personality traits that fitted them best from a list and included their reasoning. Next was an Immersive Tendencies questionnaire adapted from Schubert et al. (2001). The “dispositional awe and compassion” questionnaire was adapted from the DPES compassion and awe items (Shiota et al., 2007a). The questionnaires were translated to Dutch, reduced to around six items per questionnaire, and simplified for better comprehension by the children (van Kesteren et al., 2003). They were validated using an online questionnaire taken by 43 Dutch native speakers (age: *M* = 32.4, SD = 14.79) who answered both the original and the new questions after an immersive experience, in random order. The new Immersive Tendencies questionnaire had a reliability score of Cronbach’s α = 0.70. The original questionnaire and the children’s questionnaire correlated strongly, *r* = 0.73, *p* < 0.001, using Pearson’s correlations. The original dispositional awe and compassion questionnaire and children’s questionnaire correlated strongly as well, *r* = 0.78, *p* < 0.001, as did the subscales of the questionnaire, dispositional compassion, *r* = 0.69, *p* < 0.001, and dispositional awe, *r* = 0.64, *p* < 0.001. The newly adopted questionnaire thus measured the same constructs. The dispositional awe items had a reliability score of Cronbach’s α = 0.83 and the dispositional compassion items had α = 0.75.

The knowledge test was developed by the SpaceBuzz educational program to check for knowledge acquired in the lessons and consisted of 15 multiple-choice questions. From these 15 questions, eight questions in the pre-test matched the questions in the post-test content. To avoid a mismatch in questions in pre- and post-tests, only the matching questions were used for analysis.

#### Flight

**FIGURE 1:**
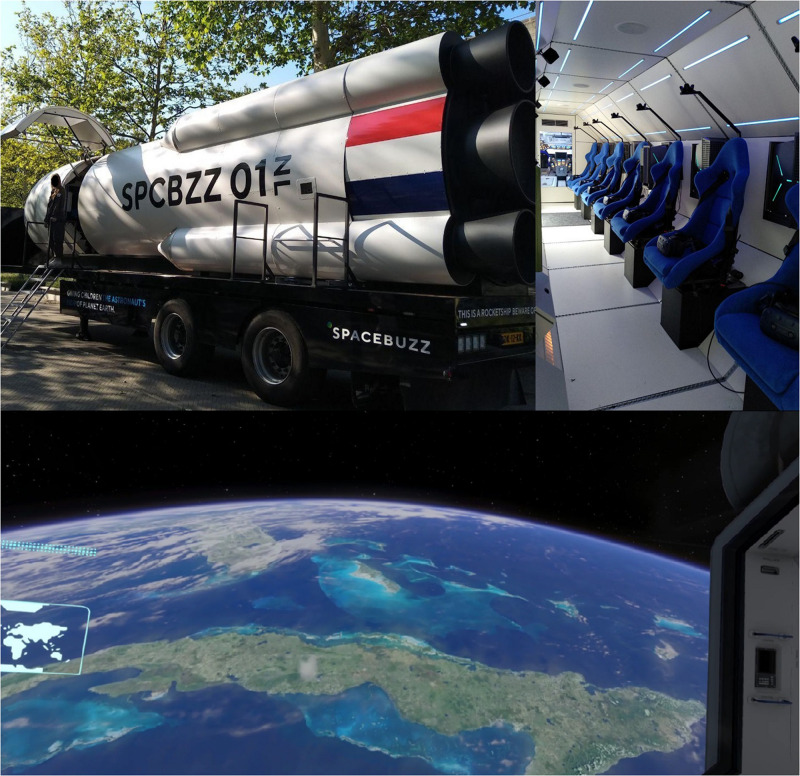
The SpaceBuzz experience. **(A)** Exterior of the SpaceBuzz rocketship. **(B)** Interior of the SpaceBuzz rocketship. **(C)** Snapshot of virtual reality experience.

The SpaceBuzz VR experience resembles a rocket ship on the back of a trailer (Figure 1A). It is 13 ft high, 8 ft wide, and 50 ft long. It has a futuristic interior and contains nine moving chairs (rotate and tilt). HTC VIVE Pro headsets were used for the VR simulations (resolution: 1,440 × 1,600 pixels per eye, 615 PPI, 3D Spatial Audio, refresh rate of 90 Hz) (Figure 1B). The simulation was created in Unity to emulate a journey to space with a length of 14 min and 25 s and recorded the participants’ gaze direction. ESA astronaut André Kuipers narrates the VR experience as the ship’s captain and is shown at the front of the rocket ship. After launching, the rocket orbits the Earth, while topics such as deforestation, excessive fishing, and pollution are discussed. After a short trip to the moon, the rocket “returns to Earth.” Head direction was recorded as a measure of attention by recording the angle between the head direction and the center of the area of interest (Figure 1C).

#### Post-flight

Immediately after experiencing the VR, another set of questionnaires was administered—the Presence questionnaire adapted from Witmer and Singer (1998). The Dutch version was simplified, shortened, and evaluated. It had a reliability score of α = 0.80. The original questionnaire and the new questionnaire correlated strongly, *r* = 0.89 *p* < 0.001. The emotions questionnaire rates feelings of awe, happiness, boredom, excitement, fear, and nausea on a five-point scale (Rudd et al., 2012; Piff et al., 2015). The emotions questions were used as a manipulation check. Nausea was added to control for simulator sickness. The dispositional awe and dispositional compassion and the personality questionnaires from pre-flight were repeated. The overview effect questionnaire contained nine questions that tap the attitude toward the planet and prosocial behavior. The knowledge test consisted of 10 multiple choice questions, where eight questions matched the questions from pre-flight in content and are thus comparable. The questions were reviewed by the creator of the first questionnaire for a good match. To control for a learning effect being caused by the pre-test and to control for pre-test results in the analysis so that low pre-test scores do not necessarily yield higher learning gains, we made sure that the matching questions only matched on topic and were not identical.

After the VR experience, the schools covered the last six lessons while back in their own classroom, within the week following their VR experience. These lessons included the final post-test questionnaires where dispositional awe and dispositional compassion, the overview effect, and personality were repeated.

### Statistical Analysis

Analyses were conducted using IBM SPSS Statistics (version 24) for calculating general results. Data collected in paper questionnaires were processed with the help of research assistants. A structural equation model (SEM), using unstandardized residuals (β), was calculated with the IBM SPSS Amos 24.0 statistical package. Questionnaires corresponding to the theorized model were used. For learning gains, proportional learning gains were calculated following Craig et al. (2004) for the eight matched questions in pre- and post-flight to account for differences in pre-test scores, avoiding bias for children that start with higher scores. Gender was also included in the SEM. In the second iteration of the SEM, described in this paper, gaze direction angle was included. This is the angle between the head direction and the center of the area of interest, which is Earth as it first comes into view until it fills the visual field. A smaller gaze angle indicates a strong focus on a specific point, on Earth. This moment is 1 min and 5 s long (for correlations, see Table 1). Maximum likelihood was used in the SEM, and missing values were handled using means and intercept estimates.

**TABLE 1:** Correlations for items that are included in the structural equation model.

Observed variable	1	2	3	4	5	6	7	8	9
1. Immersive tendencies	1								
2. Presence	0.10	1							
3. Dispositional awe	0.21**	0.18*	1						
4. Dispositional compassion	0.06	0.15*	0.37**	1					
5. Overview effect	0.11	0.27**	0.58**	0.39**	1				
6. Gender	–0.02	0.11	0.15	0.15	0.19*	1			
7. Age	–0.01	–0.05	–0.08	–0.05	−0.24**	–0.11	1		
8. Learning gains	−0.24**	0.02	0.11	–0.07	0.16	–0.09	0.10	1	
9. Gaze direction angle	0.16*	–0.12	–0.02	–0.09	−0.16*	−0.18*	0.11	–0.10	1

*Double asterisks indicate a significant correlation at the 0.01 level (two-tailed), and a single asterisk indicates a significant correlation at the 0.05 level (two-tailed).*

## Results and Discussion

Of the 188 participants who were entered for data analysis, five participants were removed because of extreme outliers in the learning gains (2.7% of proportional learning gain scores were over 3 SD away from the median), keeping the data of 183 participants for further analysis.

## General Results

The reliability of the questionnaires was assessed by computing Cronbach’s α for interrater reliability. The reliability was at an acceptable level, with Cronbach’s α of around 0.6 or higher (dispositional compassion α = 0.56, dispositional awe α = 0.70, overview effect α = 0.66). The results showed a successful manipulation of awe and the overview effect using the VR simulation. Both the average awe score *M* = 4.49 (SD = 0.72) on a five-point scale, *t*(178) = 27.48, *p* < 0.001, and the overview effect score *M* = 3.58 (SD = 0.55) on a five-point scale, *t*(179) = 14.27, *p* < 0.001, were significantly different from neutral (three on the five-point scale), demonstrating higher scores for the manipulation than for the neutral condition.

A regression analysis showed a trend in learning gains partially accounting for the overview effect, *F*(1,145) = 3.65, *p* = 0.058, *R*^2^ = 0.03. This trend can be explained by individual differences. When a median split was conducted on the pre-test scores, children scoring lower on their pre-test as determined by this split demonstrated learning gains, *F*(1,93) = 9.10, *p* = 0.003, *R*^2^ = 0.09, whereas children who scored high on their pre-test did not.

Head gaze showed a correlation between scores on the overview effect questionnaire and the average angle of gazing at Earth from space, *r* = −0.16, *p* = 0.031. This is in line with our prediction that a smaller angle of gaze, thus a more focused look toward the Earth, leads to a higher overview effect score. Additionally, angle was significantly correlated with gender, *r* = −0.18, *p* = 0.018. The difference between girls (angle: *M* = 16.73, SD = 5.50), and boys (angle: *M* = 18.46, SD = 5.45) was significant, *t*(173) = 2.08, *p* = 0.039.

### Structural Equation Model

**FIGURE 2:**
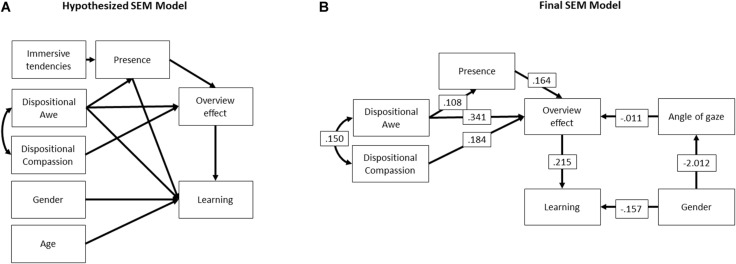
**(A)** Hypothesized structural equation model (SEM). **(B)** Final structural equation model.

Our self-report data were combined in the hypothesized model that can be found in Figure 2A. The hypothesized model did not have an acceptable fit [comparative fit index (CFI) = 0.80, Tucker–Lewis index (TLI) = 0.60, root mean square error of approximation (RMSEA) = 0.09]. We therefore iteratively removed the non-significant paths, based on the greatest misfit, until a proper model fit was reached. The final simplified model for self-report data reached an acceptable fit (CFI = 0.96, TLI = 0.89, RMSEA = 0.06). We added gaze direction to the model as a predictor for the overview effect. This model, seen in Figure 2B, reached an acceptable fit (CFI = 0.96, TLI = 0.90, RMSEA = 0.05) (for model estimates, see Table 2).

**TABLE 2:** Final model estimates for the structural equation model.

	β	SE	*p*
Dispositional awe → presence	0.11	0.05	0.017
Presence → overview effect	0.16	0.07	0.018
Dispositional awe → overview effect	0.34	0.05	<0.001
Dispositional compassion → overview effect	0.18	0.07	0.005
Gender → gaze angle	–20.01	0.82	0.014
Gaze angle → overview effect	–0.01	0.01	0.047
Overview → proportional learning gains	0.22	0.10	0.031
Gender → proportional learning gains	–0.16	0.11	0.148
Dispositional compassion ←→ dispositional awe	0.15	0.03	<0.001

Immersive tendencies did not have a significant effect on presence and were removed from the self-report model. Presence did not have an effect on learning either, and this link was also removed. Presence did, however, have an effect on the overview effect questionnaire score and stayed in the final model (β = 0.16, *p* = .018), in line with our expectations.

The dispositional awe and the dispositional compassion questionnaire scores were correlated, as expected (β = 0.15, *p* < 0.001). Dispositional awe influenced how present someone felt (β = 0.11, *p* = 0.017) and it was a strong predictor of the overview effect (β = 0.34, *p* < 0.001); it did not directly influence proportional learning gains. Dispositional compassion was another predictor for the overview effect (β = 0.18, *p* = 0.005).

Age did not have an effect on learning and was removed from the model. Gender remained in the model despite not having a significant effect because an acceptable model fit was reached. It did, however, have a very strong effect on the angle of gaze. The effect of gender on learning was negative, which means that it was stronger for boys (β = −0.16, *p* = 0.148). However, girls were the ones with smaller gaze angles (β = −2.01, *p* = 0.014), so they focused more on the center of the Earth rather than looking further away or around.

The overview effect had a significant effect on proportional learning gains (β = 0.22, *p* = 0.030). This shows that the overview effect, which is strongly correlated with awe, yields learning. The overview effect can also be predicted by the angle of gaze; although the effect is small, it is significant (β = −0.01, *p* = 0.047).

In sum, these results show that the overview effect can be predicted by dispositional awe and dispositional compassion, the feeling of presence, and the mean angle of gaze when the Earth first comes into view in VR.

## General Discussion

The primary goal of this study was to investigate whether we could create the overview effect by letting children experience a virtual space flight. We measured the recreation of the overview effect with questionnaires related to commonly associated concepts, namely, a rating of the child’s awe and compassion and questionnaires related to virtual experiences, in particular, and presence. Furthermore, we measured the effectiveness of a virtual space experience for educational purposes, with a knowledge test that was embedded within a full educational program on space, of which the VR space flight was one component. Our results showed a successful manipulation of both awe and the overview effect. We found that children with lower prior knowledge learned from the overview effect that resulted from experiencing space in VR. Additionally, the feeling of presence in VR had an effect on the overview effect questionnaire score. Children’s gaze pattern in VR was also significantly correlated with the overview effect questionnaire score. A SEM represented how the afore-mentioned constructs interact.

In this study, a new simulation for viewing Earth from space was used, embedded in an educational program that replicates an astronaut’s journey to space. The SpaceBuzz simulation proved to be effective in inducing both awe and the overview effect and thus adheres to the recommendations made by Stepanova et al. (2019a). It opens doors to use this simulation for future research on the same topic, and it confirms that the visuals were sufficiently vast and beautiful and the audio was sufficiently supportive to create a feeling of awe in the participants. Within the simulation, a personal connection is made by focusing briefly on the participants’ own country, which supposedly adds to the effect (Stepanova et al., 2019b). Even though Stepanova et al. (2019a) advised that Earth-gazing is done from three different perspectives, the two that were used (from the spacecraft and from the moon, respectively) were sufficient. Future research with this simulation can determine which aspects are most important for the induction of awe and the overview effect, giving more insight into the phenomenon.

The measurement of emotions after the VR experience included a manipulation check for the feeling of awe, similar to Piff et al. (2015) and Rudd et al. (2012). However, for more accurate measurements that would ensure that children did not already feel awed by the anticipation of entering the SpaceBuzz rocketship, in future studies, emotions before the VR experience should be monitored as well. Another way to measure awe more accurately is to employ a more substantial scale such as the AWE-S scale (Yaden et al., 2018), administered in a child-friendly way.

Our results suggest that dispositional awe has an indirect effect on learning, *via* the overview effect. Previous research shows how a disposition to feel awed increases the tendency for awe and thus the openness to learn from these vast stimuli (Shiota et al., 2007b; Valdesolo et al., 2017; Gottlieb et al., 2018). One can argue that a direct effect from dispositional awe to learning would be intuitive; however, the disposition to feel awed does not give the certainty that a participant actually feels awe in every situation. Further research may show the precise link between awe, as part of the overview effect, learning, and dispositional awe, alongside other individual differences.

In line with our expectations, the feeling of presence was correlated with and had an effect on the overview effect score in our data. Unlike Alhalabi (2016) and de Back et al. (2018), but like Parong and Mayer (2018), the presence score did not directly influence learning. One can argue that a higher feeling of presence—which did influence the overview effect score, which in turn influenced learning—is an indirect effect of presence on learning. However, with the presence and immersive tendencies questionnaires having low reliability, these results do not shed much light on issues regarding the link between VR, immersive tendencies, presence, and learning.

So far, the more commonly used physiological measures for awe and the overview effect are goosebumps (Schurtz et al., 2012; Neidlinger et al., 2017; Quesnel and Riecke, 2018), brain activity, heart interbeat intervals, skin conductance, and respiration rate (Gallagher et al., 2014; Chirico et al., 2017). The current study adds gaze patterns to this list, also suggested by Stepanova et al. (2019a), which had a small yet significant effect on overview effect scores. A significant link between gender and the angle of gaze that we see in the correlations is expected as females tend to show more explorative gaze patterns compared to males (Sargezeh et al., 2019). In further research, we can combine the afore-mentioned methods and investigate which, or which combination, would be the best predictors of awe and wonder.

This study was conducted with children participants, and because of their lower attention span and different capabilities for answering questionnaires than adults, the questionnaires were reduced and simplified (van Kesteren et al., 2003). The reliability of the new questionnaires was acceptable in the pre-test that we conducted but low for some questionnaires in the actual study. Despite it being relatively common for ecologically valid studies with diverse participants to produce weak effects of experimental variables, the low reliability of our questionnaires could have been the cause of our low significance levels. Reliability and questionnaire length are a trade-off. Previous research showed a wide variety of questionnaire length and duration (Koskelainen et al., 2000; Mikropoulos and Strouboulis, 2004; Wöber-Bingöl et al., 2014; Barry et al., 2015). For future reference, an improved overview effect questionnaire that would be highly reliable, yet manageable in length, understandable, and clear for children may increase the reliability of the study and could also improve the significance of the overall results.

The findings of this study add to existing research on awe and the overview effect and show insights gained from a large number of children participants, embedded in an educational program. Both the connection between the overview effect and study performance and the link between the overview effect and gaze data open doors for both future research on these topics as well as using immersive VR experiences in educational programs (Louwerse et al., 2020), thereby creating young ambassadors of planet Earth.

## Data Availability Statement

The datasets presented in this article are not readily available because they contain sensitive data of minors. Requests to access these datasets should be directed to ML (mlouwerse@uvt.nl).

## Ethics Statement

The studies involving human participants were reviewed and approved by the Ethical Review Board of Tilburg School of Humanities and Digital Sciences, Tilburg University. Written informed consent to participate in this study was provided by the participants’ legal guardian/next of kin.

## Author Contributions

All authors contributed to the conception and design of the study, performed data gathering and statistical analysis, contributed to the manuscript and read and approved the submitted version. ML and MP received project funding.

## Conflict of Interest

The authors declare that the research was conducted in the absence of any commercial or financial relationships that could be construed as a potential conflict of interest.
